# Genetic Regulation of Alginate Production in *Azotobacter vinelandii* a Bacterium of Biotechnological Interest: A Mini-Review

**DOI:** 10.3389/fmicb.2022.845473

**Published:** 2022-03-23

**Authors:** Cinthia Núñez, Liliana López-Pliego, Carlos Leonel Ahumada-Manuel, Miguel Castañeda

**Affiliations:** ^1^Departamento de Microbiología Molecular, Instituto de Biotecnología, Universidad Nacional Autónoma de México, Cuernavaca, Mexico; ^2^Centro de Investigaciones en Ciencias Microbiológicas, Instituto de Ciencias, Benemérita Universidad Autónoma de Puebla, Puebla, Mexico

**Keywords:** alginate, *Azotobacter vinelandii*, genetic regulation, GacS/A-Rsm, c-di-GMP

## Abstract

Alginates are a family of polymers composed of guluronate and mannuronate monomers joined by β (1–4) links. The different types of alginates have variations in their monomer content and molecular weight, which determine the rheological properties and their applications. In industry, alginates are commonly used as additives capable of viscosifying, stabilizing, emulsifying, and gelling aqueous solutions. Recently, additional specialized biomedical uses have been reported for this polymer. Currently, the production of alginates is based on the harvesting of seaweeds; however, the composition and structure of the extracts are highly variable. The production of alginates for specialized applications requires a precise composition of monomers and molecular weight, which could be achieved using bacterial production systems such as those based on *Azotobacter vinelandii*, a free-living, non-pathogenic bacterium. In this mini-review, we analyze the latest advances in the regulation of alginate synthesis in this model.

## Introduction

Alginates are linear polysaccharides composed of varying proportions of β-d-mannuronate (M) linked by a β-1,4 bond to α-l-guluronate (G) residues. Alginates are important biopolymers with applications in the medical and industrial fields, where they are used as stabilizing agents, thickeners, and gelling agents. Alginate microspheres have been used in the therapeutic administration for the controlled release of drugs, proteins, vaccines, and cells (Dhamecha et al., 2019). Currently, alginate is obtained from brown algae. However, the composition of the polymer varies according to environmental conditions. The production of alginates that are useful for specialized applications requires particular physicochemical properties, which is difficult to achieve using seaweeds as a production source (Remminghorst and Rehm, 2006; Hay et al., 2013; Urtuvia et al., 2017). The genus *Pseudomonas* and *Azotobacter* also produce this polymer as an exo-polysaccharide (Hay et al., 2013; Urtuvia et al., 2017).

In the opportunistic pathogen *Pseudomonas aeruginosa*, alginate is important for the formation of bacterial communities that grow embedded in an exo-polysaccharide matrix and adhered to an inert surface or living tissue, better known as biofilms, providing a thick protective layer against the host immune system and antimicrobial agents (Leid et al., 2005; Lovewell et al., 2014). In the free-living bacterium *Azotobacter vinelandii*, alginate is produced in large quantities during its vegetative growth, where it serves as a barrier against the diffusion of heavy metals and oxygen (Sabra et al., 2001).

The main difference between alginates from algae and bacteria resides in the acetylation of M residues in positions 2 and 3 (C2 and C3; Hay et al., 2013). The presence of two monomeric units (M and G) in the alginate chain allows different distributions of them. The formation of M blocks, characteristic for the presence of consecutive M residues, has been observed, as well as G blocks and MG blocks, with alternating M and G residues. The presence of these blocks as well as the degree of acetylation and molecular weight (MW) of alginates have a strong impact on the rheological properties of alginate solutions (Lee and Mooney, 2012; Urtuvia et al., 2017). *Azotobacter vinelandii* has been proposed as a bacterial source for the production of alginates with defined composition (Hay et al., 2013; Urtuvia et al., 2017). In this mini-review, we analyze the latest advances in the regulation of alginate synthesis in this model.

## Alginate Biosynthesis

**Figure 1:**
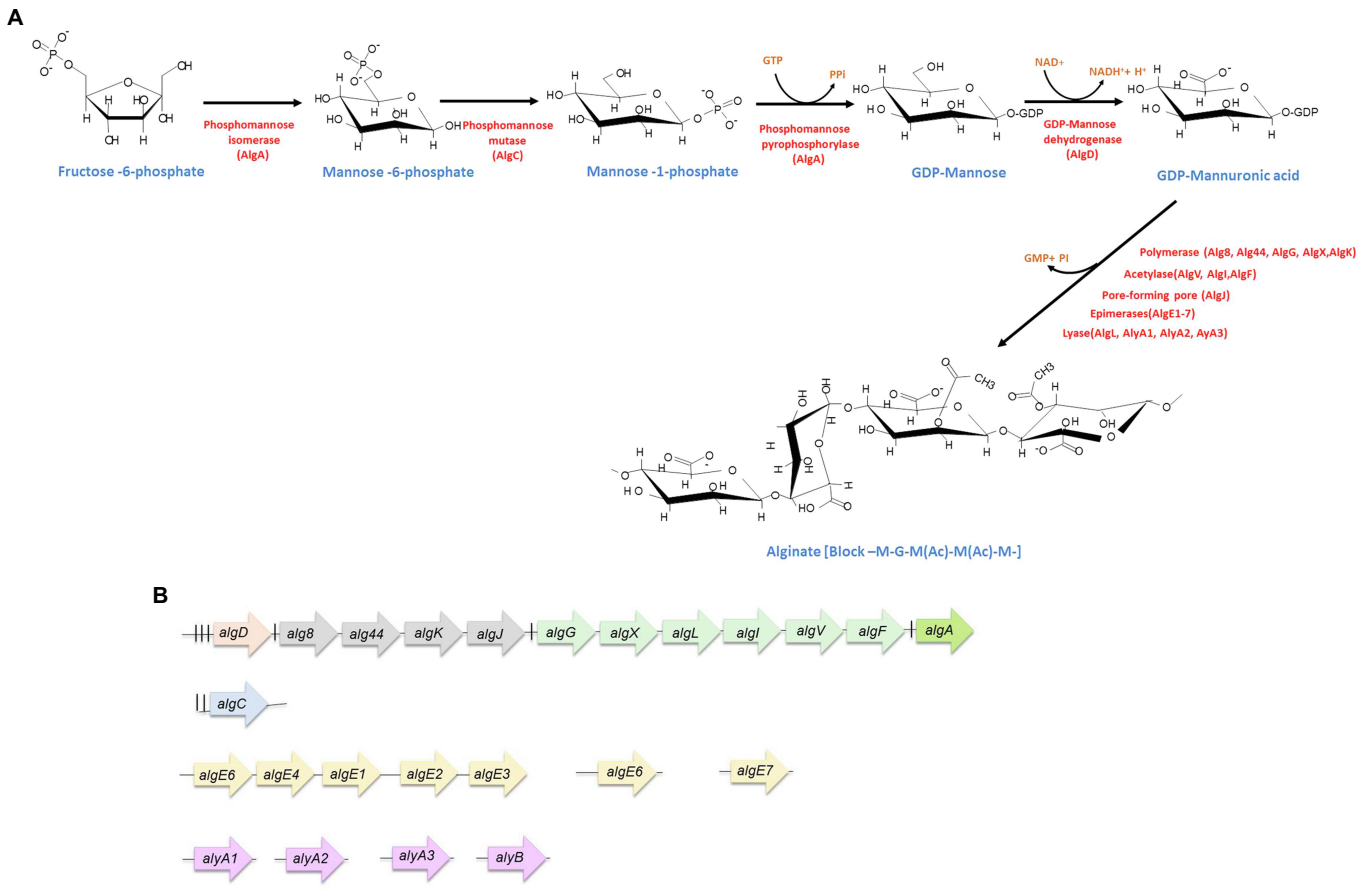
Alginate synthesis in *Azotobacter vinelandii*. **(A)** Biosynthetic pathway. The enzymes (red) as well as the substrates (blue) for alginate production are indicated. **(B)** Genetic arrangement of alginate biosynthetic genes. The experimentally determined promoters are indicated by vertical black lines. *algD* transcription starts from three promoters (for details, see the text). The nature of the internal promoters upstream *alg8, algG*, and *algA* is unknown. Genes are not shown at scale.

The alginate biosynthesis process is highly conserved in *A. vinelandii* and *P. aeruginosa* (Franklin et al., 2011; Hay et al., 2013; Urtuvia et al., 2017). Most of the alginate biosynthetic genes (*alg*) are grouped in a region of the chromosome and are headed by *algD*; only *algC* resides in a different *locus*. This cluster (*algD-8-44-K-J-X-L-I-V-F-A*) contains genes for the generation of the monomer (*algD* and *algA*); for polymerization and transfer through the inner membrane (*alg8* and *alg44*); for periplasmic transfer and modification (*algK*, *algG*, *algX*, *algL, algI, algV*, and *algF*); and for export through the outer membrane (*algJ*; Hay et al., 2014; Figure 1).

Alginate synthesis begins with the synthesis of fructuse-6-phosphate which is the substrate of the cytosolic enzymes phosphomannose isomerase/GDP-mannose pyrophosphorylase (encoded by *algA*), phosphomanomutase (encoded by *algC*), and GDP-mannose dehydrogenase (encoded by *algD*); this last enzyme catalyzes an irreversible reaction that produces the activated monomer GDP-mannuronic acid. This oxidation reaction is the key limiting reaction and compromises carbon flux toward alginate synthesis (Remminghorst and Rehm, 2006). The GDP-mannuronic acid is then polymerized by the Alg8-Alg44 inner membrane complex. Alg8 is a type-2 glycosyl transferase and Alg44 is a multidomain protein, with a PilZ cytosolic domain, a transmembrane region, and a periplasmic domain. The latter domain physically interacts with the other proteins that are part of the periplasmic scaffold, involved in the transit, modification, and secretion of alginate (Hay et al., 2013; Moradali et al., 2015). The nascent alginate chain (poly-M) is transported across the periplasm by the periplasmic scaffold conformed by proteins AlgG, AlgX, AlgK, and the periplasmic region of Alg44, which guides the alginate from the polymerase complex, residing in the inner membrane to the outer membrane porin AlgJ (Hay et al., 2013; Moradali et al., 2015). During this transit, the alginate chain is acetylated at its M residues by the acetyltransferase complex, formed by AlgI, AlgF, AlgV, and AlgX (Hay et al., 2013; Moradali et al., 2015). Furthermore, M residues that are not acetylated can be epimerized to G residues by the periplasmic epimerase AlgG. However, it has been shown that in *A. vinelandii*, the activity of AlgG is negligible and the epimerization of M residues to G in *A. vinelandii* is conducted by a family of extracellular mannuronan C-5 epimerases, called AlgE1-AlgE7, once the polymer has been secreted to the medium (Hoidal et al., 2000; Gimmestad et al., 2006; Hay et al., 2013; Ertesvåg, 2015; Figure 1).

*algL* encodes an alginate lyase, whose activity is to degrade the alginate chain that mistakenly fails to be secreted by the outer membrane porin (Bakkevig et al., 2005; Hay et al., 2013). Besides AlgL, *A. vinelandii* encodes another five alginate lyases, AlyA1, AlyA2, AlyA3, AlyB, and AlgE7, the latter is an enzyme with dual lyase/epimerase activity and belongs to the family of extracellular C-5 epimerases mentioned above (Ertesvåg, 2015; Figure 1A); only AlgL has been shown to affect the alginate chain length of the secreted polymer (Trujillo-Roldán et al., 2003).

## Genetic Regulation of Alginate Synthesis

Even though most of the structural genes involved in alginate synthesis share a high degree of identity between *P*. *aeruginosa* and *A. vinelandii*, the regulation does have marked differences. In *P. aeruginosa*, the main *alg* gene cluster was originally described to conform an operon headed by *algD*, the transcription of which is started from a single AlgU-dependent promoter (Chitnis and Ohman, 1993). In *A. vinelandii*, however, the *alg* genes are arranged in three operons (Figure 1B), the first one containing the *algD* gene as the only element (Campos et al., 1996; Figure 1B). Weak internal promoters in the *P. aeruginosa alg* cluster have been reported, resulting in transcriptional units similar to those described for *A. vinelandii* (Paletta and Ohman, 2012). In *A. vinelandii*, additional alginate modifying genes, i.e., *algE1-7*, encoding mannuronan C-5 epimerases or those encoding alginate lyases, are located in different regions of the chromosome (Gimmestad et al., 2009; Ertesvåg, 2015; Figure 1B).

## Transcriptional Regulation

**Figure 2:**
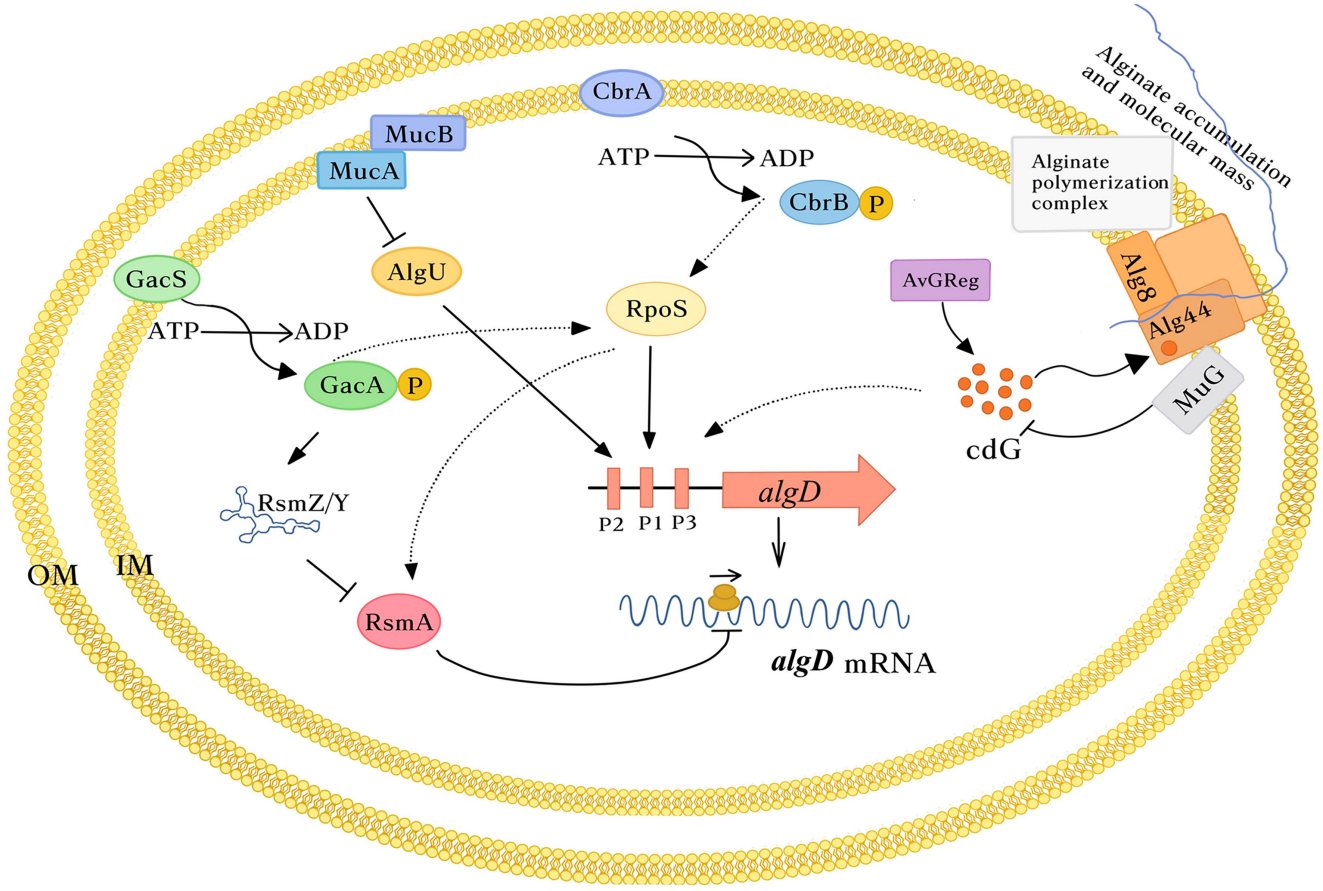
Schematic representation of *algD* regulation in *Azotobacter vinelandii*. Transcription of *algD* starts from three promoters that depends on the stress response sigma factor AlgU and the stationary phase sigma factor RpoS. The two-component systems CbrA/B and GacS/A exert a positive effect on RpoS. *algD* translation is under the control of the GacS/A-Rsm system. GacA activates transcription of a family of sRNAs of the RsmY/Z family counteracting RsmA activity. This last protein directly binds *algD* mRNA blocking translation. The c-di-GMP control module, composed of the DGC AvGReg and the PDE MucG, modulates la pool of this second messenger presumably in the vicinity to the Alg8-Alg44 polymerase complex, regulating the amount and the MM of the polymer produced. The c-di-GMP levels positively influence *algD* transcription. For simplicity, only one *algD* transcriptional unit is shown. In the periplasmic scaffold, responsible for alginate transport and secretion, only the polymerase complex Alg8-44 is shown. Dashed arrows, indirect effect.

Since *algD* encodes the key enzyme in the alginate biosynthetic pathway, its expression is finely controlled, both at the transcriptional and post-transcriptional levels (Figure 2). In *A. vinelandii*, *algD* transcription is initiated from three promoters, which are recognized by the stationary phase sigma factor RpoS (σ^s^; P1*algD*) and the stress response sigma factor AlgU (σ^E^; P2*algD*). The nature of P3*algD* remains unknown (Campos et al., 1996; Núñez et al., 2000a; Castañeda et al., 2000, 2001; Figure 2).

*Azotobacter vinelandii* AlgU is also essential for alginate synthesis as it is necessary for the expression of *algC* (Gaona et al., 2004). As in *Escherichia coli* and *P. aeruginosa*, the activity of the sigma factor AlgU is antagonized by the anti-sigma factors MucA and MucB (Martínez-Salazar et al., 1996). In this way, alginate production is impaired in an *algU* mutant, while in a *mucAB* mutant, it increases, due to higher levels of *algD* transcription (Moreno et al., 1998; Núñez et al., 2000a). However, unlike *A. vinelandii*, alginate accumulation in *P. aeruginosa* is triggered only under conditions increasing the activity of AlgU, such as in clinical isolates carrying *mucA* mutations or upon cell envelope stress, that triggers a regulated intra-membrane proteolysis of MucA, releasing the sigma factor AlgU and directing the RNA polymerase to activate *algD* (Hay et al., 2014). Of note, impairment of cell wall recycling in *A. vinelandii* also enhances alginate production by increasing *algD* transcription, presumably through a conserved AlgU-dependent mechanism (Núñez et al., 2000b).

In *P. aeruginosa*, the expression of *algD* is directly regulated by the transcriptional factors AlgR, AlgB, and AmrZ (initially called AlgZ; Ma et al., 1998; Leech et al., 2008; Jones et al., 2014; Kong et al., 2015; Xu et al., 2016). In *A. vinelandii* however, AlgR does not affect *algD* transcription (Núñez et al., 1999). In the case of AlgB, a recent study by Mærk et al. found that a mutation in *algB* did not affect alginate production, which is in sharp contrast to its role in *P. aeruginosa* (Leech et al., 2008; Mærk et al., 2020). In the same study, it was found that the AmrZ regulator (Alginate and Motility Regulator) is necessary for alginate production in *A. vinelandii*, since an *amrZ*^−^ mutant reduces the production of the polymer, but the molecular mechanism of this regulation remains to be elucidated (Mærk et al., 2020).

## Post-transcriptional Regulation

In *A. vinelandii*, the two-component system GacS/A is involved in the control of alginate synthesis (Figure 2). The effect of GacS/GacA is mediated by the RsmA/RsmZY post-transcriptional regulatory system (Castañeda et al., 2000, 2001, 2016). RsmA is a protein that binds directly to the *algD* mRNA, preventing its translation. GacA activates the transcription of a family of small regulatory sRNAs (RsmZ1-7 and RmsY), which antagonize RsmA activity by titrating this protein, allowing *algD* mRNA translation (Manzo et al., 2011; Hernandez-Eligio et al., 2012; López-Pliego et al., 2018). Thus, a *gacA* mutation impairs *rsm*-sRNAs transcription and totally abrogates *algD* expression and consequently, alginates synthesis (Castañeda et al., 2001). In contrast, the effect of individual *rsm*-sRNAs mutations on alginate synthesis differs among them. Interestingly, transcriptional profiles of the *rsm*-sRNAs show a differential expression pattern but it does not correlate with the alginate phenotype observed for each *rsm*-sRNA mutant, suggesting the existence of additional unknown factors affecting their activity (López-Pliego et al., 2018, 2020). In *P. aeruginosa*, the GacS/A-Rsm pathway has not been reported to regulate alginate biosynthesis (Hay et al., 2014).

Another two-component system involved in alginate synthesis in *A. vinelandii* is CbrA/CbrB that heads a regulatory cascade controlling carbon catabolic repression in this bacterium (Quiroz-Rocha et al., 2017); similar to GacS/A, the CbrA/B homologue system in *P. aeruginosa* has not been reported to control alginate production. The observed negative effect of CbrA/B is related to the Rsm system. CbrA/B is necessary to reach high expression levels of the *rsmA* gene and accordingly, in a CbrA-deficient mutant, there exists a de-repression of *algD* translation. CbrA/B exerted a positive effect on RpoS accumulation partially explaining the positive control of *rsmA* expression by this system, as one of the promoters directing *rsmA* expression is RpoS-dependent. To date, the mechanism of *rsmA* regulation by CbrA/B is unknown (Quiroz-Rocha et al., 2017).

## Post-translational Regulation

Cyclic bis-(3′, 5′)-guanosine monophosphate, c-di-GMP, is a second messenger that regulates a large number of cellular processes, including exo-polysaccharide production (Jenal et al., 2017). It is synthesized by diguanylate cyclases (DGC) and degraded by phosphodiesterases (PDE). To exert its effect, c-di-GMP must bind to effector molecules. To date, many effectors have been described, such as proteins with PilZ domains (Chou and Galperin, 2016). The alginate biosynthetic process was first related to c-di-GMP when the PilZ domain was identified at the C-terminal end of the Alg44 co-polymerase in *P. aeruginosa* (Amikam and Galperin, 2006). Binding of c-di-GMP to the Alg44 PilZ domain is essential to activate the alginate polymerase complex Alg8-Alg44 (Merighi et al., 2007). In *P. aeruginosa*, the inner membrane protein MucR is responsible for c-di-GMP synthesis in the vicinity of Alg44 PilZ (Hay et al., 2009).

In *A. vinelandii*, c-di-GMP exerts a positive control on alginate biosynthesis. Artificially generated high or reduced levels of this second messenger boosted or impaired, respectively, the production of this polymer. In *A. vinelandii*, the *Av*GReg protein, but not MucR, is the DGC providing the c-di-GMP necessary for alginate polymerization (Ahumada-Manuel et al., 2020). Furthermore, the inner membrane protein MucG was identified as the only PDE inhibiting alginate production. MucG is a multidomain signaling protein that besides carrying both, c-di-GMP synthesis (GGDEF) and degradation (EAL) domains contain a PAS domain, involved in sensing the intracellular redox status. A MucG-deficient mutant exhibits increased c-di-GMP concentration and alginate production, relative to the wild-type strain (Ahumada-Manuel et al., 2017, 2020). Interestingly, in *A. vinelandii*, the c-di-GMP also exerts a positive effect on the alginate chain length. High levels of this second messenger favor the production of high molecular-mass (MM) alginate and this effect is not strain specific.

In *A. vinelandii*, the MM of the polymer is regulated by the oxygen transfer rate (OTR) in the culture medium (Flores et al., 2014); a reduction in the maximum OTR increases the MM of the alginate, correlating with a simultaneous increase in the pool of c-di-GMP. However, the MucG-deficient mutant produces high MM alginate independently of the OTR in the culture medium with respect to the wild-type strain. MucG has a PAS domain, predicted to bind the FMN cofactor, sensing the intracellular redox state. Therefore, the PDE activity of MucG seems to be regulated by differences in the redox state, determining the c-di-GMP pool in the vicinity of Alg44 and consequently the alginate MM (Ahumada-Manuel et al., 2020). Regulation by c-di-GMP of alginate modification in *A. vinelandii* is not restricted to the control of the alginate MM as expression of the AlgE1-6 mannuronan C-5 epimerases is under the positive control of c-di-GMP. The G-rich alginate chains produced by the activity of these enzymes are essential for the correct assembly of the alginate envelope that protects *A. vinelandii* differentiated cells, called cysts (Martínez-Ortiz et al., 2020). The control by c-di-GMP of the AlgE1-6 enzymes under vegetative growth deserves to be investigated.

## Metabolic Effects on Alginate Production

Alginate production is an energy and carbon demanding pathway finely regulated by the metabolic status of the cell. Mærk et al. (2020) showed that in general, the synthesis of alginate requires an optimum metabolism, as disruption of genes involved in biosynthetic pathways affecting the production of vitamins, purines, or tricarboxylic acid cycle intermediates, among others, reduces or abolishes the production of this polymer. Accordingly, supplementing the culture medium with some of these compounds favors alginate production. *Azotobacter vinelandii* produces the intracellular polyester poly-β-hydroxybutyrate (PHB) that competes with alginate by the carbon source. As expected, mutations blocking PHB production favor the synthesis of alginate due to higher carbon source availability (Segura et al., 2003). However, the absence of PHB causes an imbalance in the intracellular redox state negatively impacting cell growth (Jiménez et al., 2016). Other *loci* positively influencing alginate production were identified and include *fruA* (Mærk et al., 2020). This gene encodes a protein belonging to a fructose phosphoenolpyruvate phosphotransferase system (PTS^Fru^). *fruA* mutants do not produce alginate. This phenotype is expected, when fructose is used as the sole carbon source, but interestingly, this effect is maintained in gluconeogenic carbon sources, suggesting that FruA, in addition to control sugar assimilation, exerts other regulatory functions during alginate synthesis. The *fruA* phenotype synthesis was proposed to be derived from a crosstalk between the PTS^Fru^ and PTS^Ntr^ systems as has been reported in *Pseudomonas putida* (Chavarría et al., 2013). However, this is unlikely as alginate production is not affected in mutants deficient in the PTS^Ntr^ system (Trejo et al., 2017).

The respiratory activity of *A. vinelandii* has been shown to influence alginate synthesis. This strict aerobic bacterium is characterized by its high respiratory capacity (Dalton and Postgate, 1968). Three different NADH dehydrogenases transfer reducing equivalents to a common pool of ubiquinone-8 (Q_8_; Bertsova et al., 1998, 2001). A reduction in the transcription of *ubiA*, encoding a Q_8_ biosynthetic enzyme, decreases the respiratory activity but increases alginate production (Núñez et al., 2013). Similarly, the absence of the Na+-translocating NADH:ubiquinone oxidoreductases, a redox-driven sodium pump, enhances the levels of alginate (Núñez et al., 2009), indicating a regulatory link between a transmembrane sodium gradient and alginate synthesis in the cell.

## Conclusion

In despite the close phylogenetic relationship between *P. aeruginosa* and *A. vinelandii*, the regulation of alginate production in these two bacteria has marked differences, likely derived from their different habitats and roles that this polysaccharide plays in these organisms. To date, there exists a vast knowledge about the genetics and the metabolic/environmental aspects controlling the production of alginate by *A. vinelandii*. These studies have been mainly motivated by the biotechnological potential of this bacterium to be used as a source for the production of alginate, as in contrast to *P. aeruginosa*, *A. vinelandii* is considered a GRAS (generally recognized as safe) microorganism.

The current knowledge about the complex regulatory network controlling alginate production in *A. vinelandii* at the transcriptional, post-transcriptional, and post-translational level sets this bacterium as a promising source for the synthesis of tailor-made polymers. In fact, this knowledge has already allowed the designing of particular genetic modifications combined with bioengineering strategies to improve both, alginate production yields and the structural characteristics of the polymer. An example of this is the construction of strains lacking the PDE MucG or the alginate lyase AlgL, grown under conditions of low oxygen concentration (Trujillo-Roldán et al., 2003; Ahumada-Manuel et al., 2020). Since oxygen levels influence the expression of several biosynthetic genes, much of the effort to improve alginate production has been focused on this factor (Flores et al., 2014). There are still some regulators that modify the production of alginates that could be tested in the already established culture conditions or in novel conditions, combining mutations, and/or over-expression of relevant genes that are known to impact the production of this polymer. Therefore, a better understanding of all the regulatory aspects of this process is important not only for optimizing the bacterial biosynthesis of this polymer but for producing alginate chains of defined compositions and particular physicochemical traits.

## Author Contributions

MC and CN conceived, designed, edited, and revised the manuscript. CN, MC, LL-P, and CA-M conducted the literature study and wrote the draft manuscript. All authors contributed to the article and approved the submitted version.

## Funding

This work was supported by VIEP-BUAP, grant 100301900-VIEP2021 to MC, and by project PAPIIT-DGAPA, UNAM IN-209521 to CN.

## Conflict of Interest

The authors declare that the research was conducted in the absence of any commercial or financial relationships that could be construed as a potential conflict of interest.

## Publisher’s Note

All claims expressed in this article are solely those of the authors and do not necessarily represent those of their affiliated organizations, or those of the publisher, the editors and the reviewers. Any product that may be evaluated in this article, or claim that may be made by its manufacturer, is not guaranteed or endorsed by the publisher.
