# Corrigendum: Genetic Regulation of Alginate Production in *Azotobacter vinelandii* a Bacterium of Biotechnological Interest: A Mini-Review

**DOI:** 10.3389/fmicb.2022.940908

**Published:** 2022-06-09

**Authors:** Cinthia Núñez, Liliana López-Pliego, Carlos Leonel Ahumada-Manuel, Miguel Castañeda

**Affiliations:** ^1^Departamento de Microbiología Molecular, Instituto de Biotecnología, Universidad Nacional Autónoma de México, Cuernavaca, Mexico; ^2^Centro de Investigaciones en Ciencias Microbiológicas, Instituto de Ciencias, Benemérita Universidad Autónoma de Puebla, Puebla, Mexico

**Keywords:** alginate, *Azotobacter vinelandii*, genetic regulation, GacS/A-Rsm, c-di-GMP

In the original article, there was a mistake in Figure 1A as published. The names of the third and fourth enzymes of the alginate synthesis pathway are wrong. The correct name for the third enzyme is GDP-mannose pyrophosphorylase (AlgA), and the correct name for the fourth enzyme is GDP-mannose dehydrogenase (AlgD). The corrected figure appears below.

**Figure 1:**
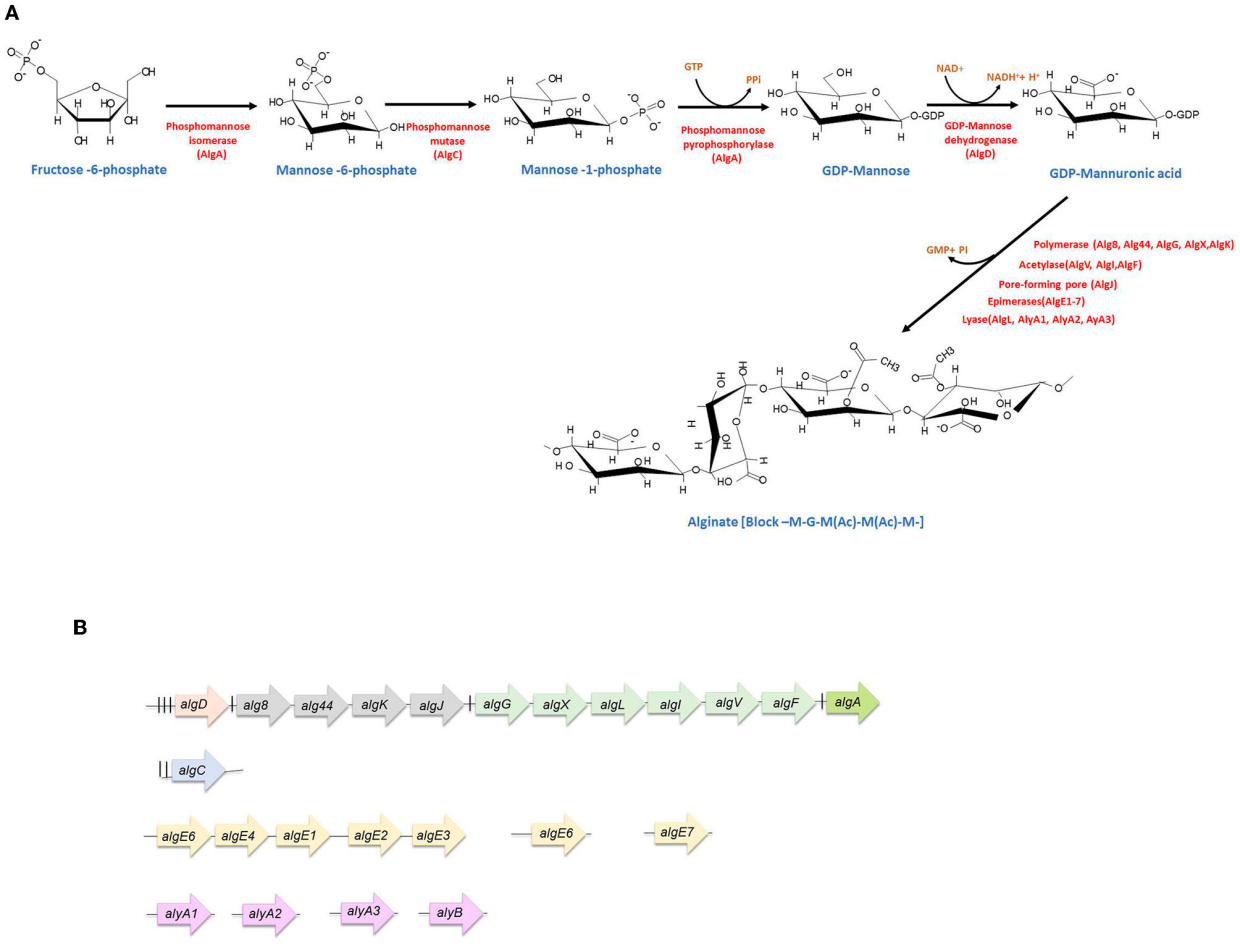
Alginate synthesis in *Azotobacter vinelandii*. **(A)** Biosynthetic pathway. The enzymes (red) as well as the substrates (blue) for alginate production are indicated. **(B)** Genetic arrangement of alginate biosynthetic genes. The experimentally determined promoters are indicated by vertical black lines. *algD* transcription starts from three promoters (for details, see the text). The nature of the internal promoters upstream *alg8, algG*, and *algA* is unknown. Genes are not shown at scale.

The authors apologize for this error and state that this does not change the scientific conclusions of the article in any way. The original article has been updated.

## Publisher's Note

All claims expressed in this article are solely those of the authors and do not necessarily represent those of their affiliated organizations, or those of the publisher, the editors and the reviewers. Any product that may be evaluated in this article, or claim that may be made by its manufacturer, is not guaranteed or endorsed by the publisher.

