# Supplementary material for: Genetic Regulation of Alginate Production in Azotobacter vinelandii a Bacterium of Biotechnological Interest: A Mini-Review
Source: Front Microbiol. 2022 Mar 23;13:845473. doi: 10.3389/fmicb.2022.845473 (PMC8988225; doi:10.3389/fmicb.2022.845473)
Supplement: Supplementary file 1 [file Data_Sheet_1.PDF]

Table S1. Description of the biosynthetic *alg* genes in *A. vinelandii.*

Gene	Locus tag	DNA Coordinates	Encoded Protein	Activity	Regulators	Promoter(s)	Mutant phenotype*	References
algD	Avin_10970<br>AVIN_RS04990	1049330..1050640 (-)	AlgD	GDP-mannose 6-dehydrogenase	RpoS (σ^S), AlgU (σ^E), MucABDC, GacS/A-Rsm, CbrA/B	*algD* p1 (not defined), *algDp* 2 (σS)<br>*algD* p3 (σ^E)	Unable to produce alginate	Moreno et al., 1998; Núñez et al., 2000; Castañeda et al., 2001;
alg8	Avin_10960<br>AVIN_RS04985	1047735-1049219 (-)	Alg8	Glycosyltransferase/ polymerase		*Alg8* p1	Unable to produce alginate	Mejía-Ruíz et al., 1997a
alg44	Avin_10950<br>AVIN_RS04980	1046532..1047698 (-)	Alg44	Alginate copolymerase	AvGReg, MucG		Unable to produce alginate	Mejía-Ruíz et al., 1997a Ahumada-Manuel et al 2020
algK	Avin_10940<br>AVIN_RS04975	1045145..1046518 (-)	AlgK	alginate biosynthesis protein AlgK			Unable to produce alginate	Mejía-Ruíz et al., 1997b
algJ	Avin_10930<br>AVIN_RS04970	1043646..1045136 (-)	AlgJ	Alginate export porin			ND	Mejía-Ruíz et al., 1997b
algG	Avin_10920<br>AVIN_RS04965	1042036..1043616 (-)	AlgG	Mannuronan C-5-Epimerase		*algG* p1 (not defined)	ND	Mejía-Ruíz et al., 1997b, Vazquez et al., 1999
algX	Avin_10910<br>AVIN_RS04960	1040564..1042015 (-)	AlgX	Alginate O-acetyltransferase			ND	Vazquez et al., 1999
algL	Avin_10900<br>AVIN_RS04955	1039431..1040555 (-)	AlgL	Alginate lyase			Increased alginate molecular weight	Trujillo-Roldán et al 2000…)
algI	Avin_10890<br>AVIN_RS04950	1037739..1039238 (-)	AlgI	Alginate O-acetyl transferase			ND	Vazquez et al., 1999
algV	Avin_10880<br>AVIN_RS04945	1036560..1037726 (-)	AlgV	Alginate O-acetyltransferase			ND	Vazquez et al., 1999

algF	Avin_10870 AVIN_RS04940	1035871..1036524 (-)	AlgF	Alginate O-acetyltransferase			Production of non-acetylated alginate	Vazquez et al., 1999
algA	Avin_10860 AVIN_RS04935	1034329..1035774 (-)	AlgA	Phosphomannose isomerase/GDP-mannose pyrophosphorylase			Unable to produce alginate	Segura et al., 2003
algC	Avin_02910 AVIN_RS01410	273820-275217 (-)	AlgC	Phosphomannose mutase	AlgU(σ^E)	*algC* p1 (σ^E) and *algC* p2 (not defined)	Unable to produce alginate	Gaona et al., 2004
alyA1	Avin_31810 AVIN_RS14535	3289398..3290111 (-)	AlyA1	Alginate lyase			Apparently reduced alginate production	Gimmestad 2009
alyA2	Avin_23960 AVIN_RS10955	2395224..2395946 (+)	AlyA2	Alginate lyase			ND	Gimmestad 2009
alyA3	Avin_13810 AVIN_RS06280	1341827..1343080 (+)	AlyA3	Alginate lyase			ND	Gimmestad 2009
algE1	Avin_51190 AVIN_RS23410	5194329..5198540 (-)	AlgE1	Poly(beta-D-mannuronate) C5 epimerase 1	RpoS (σ^S)		No effect on alginate structure was reported	Steigedal et al., 2008; Moreno et al., 2018
algE2	Avin_51180 AVIN_RS23405	5191069..5194065 (-)	AlgE2	Poly(beta-D-mannuronate) C5 epimerase 2	RpoS (σ^S)		No effect on alginate structure was reported	Steigedal et al., 2008; Moreno et al., 2018
algE3	Avin_51170 AVIN_RS23400	5185271..5190790 (-)	AlgE3	Poly(beta-D-mannuronate) C5 epimerase 3	RpoS (σ^S)		Individual mutant synthesizes alginate with a lower G content.	Steigedal et al., 2008; Moreno et al., 2018

algE4	Avin_51200 AVIN_RS23415	5198816..5200477 (-)	AlgE4	Secreted mannuronan C-5 epimerase /Poly(beta-D-mannuronate) C5 epimerase 4	RpoS (σ^S)		No effect on alginate structure was reported	Steigedal et al., 2008; Moreno et al., 2018
algE5	Avin_33710 AVIN_RS15320	3445248..3448244 (-)	AlgE5	Poly(beta-D-mannuronate) C5 epimerase 5	RpoS (σ^S)		No effect on alginate structure was reported	Steigedal et al., 2008; Moreno et al., 2018
algE6	Avin_51230 AVIN_RS23420	5200752..5203376 (-)	AlgE6	Poly(beta-D-mannuronate) C5 epimerase 6	RpoS (σ^S)		No effect on alginate structure was reported	Steigedal et al., 2008; Moreno et al., 2018
algE7	Avin_51250 AVIN_RS23435	5208142..5210712 (-)	AlgE7	Secreted bifunctional mannuronan C-5 epimerase/alginate lyase	RpoS (σ^S)		Impaired releasing of alginate chains from cell surface	Steigedal et al., 2008; Gimmestad 2009

* alginate phenotype of the strain carrying a knock out mutation
ND Not Determined

References

Ahumada-Manuel, C. L., Martínez-Ortiz, I. C., Hsueh, B. Y., Guzmán, J., Waters, C. M., Zamorano-Sánchez, D., Espín, G., & Núñez, C. (2020). Increased c-di-GMP Levels Lead to the Production of Alginates of High Molecular Mass in Azotobacter vinelandii. Journal of bacteriology,
.

Campos, M., Martínez-Salazar, J. M., Lloret, L., Moreno, S., Núñez, C., Espín, G., et al. (1996). Characterization of the gene coding for GDP-mannose dehydrogenase (*algD*) from *Azotobacter vinelandii* . *J. Bacteriol.* 178, 1793–1799. doi:10.1128/jb.178.7.1793-1799.1996.

Castañeda, M., Sánchez, J., Moreno, S., Núñez, C., and Espín, G. (2001). The Global Regulators GacA and σS Form Part of a Cascade That Controls Alginate Production in *Azotobacter vinelandii*. *J. Bacteriol*. 183, 6787–6793. doi:10.1128/JB.183.23.6787-6793.2001.

Gimmestad, M., Ertesvåg, H., Heggeset, T. M. B., Aarstad, O., Svanem, B. I. G., and Valla, S. (2009). Characterization of Three New *Azotobacter vinelandii* Alginate Lyases, One of Which Is Involved in Cyst Germination. *J. Bacteriol* . 191, 4845–4853. doi:10.1128/JB.00455-09.

Manzo, J., Cocotl-Yañez, M., Tzontecomani, T., Martínez, V. M., Bustillos, R., et al. (2011). Post-Transcriptional Regulation of the Alginate Biosynthetic Gene *algD* by the Gac/Rsm System in *Azotobacter vinelandii* . *MMB* 21, 147–159. doi:10.1159/000334244.

Mejía-Ruíz, H., Guzmán, J., Moreno, S., Soberón-Chávez, G., & Espín, G. (1997). The *Azotobacter vinelandii alg8* and *alg44* genes are essential for alginate synthesis and can be transcribed from an *algD* -independent promoter. *Gene* , 199(1-2), 271–277. https://doi.org/10.1016/s0378-1119(97)00380-6

Mejía-Ruíz, H., Moreno, S., Guzmán, J., Nájera, R., León, R., Soberón-Chávez, G., & Espín, G. (1997). Isolation and characterization of an *Azotobacter vinelandii algK* mutant. *FEMS microbiology letters* , 156(1), 101–106. https://doi.org/10.1111/j.1574-6968.1997.tb12712.x

Moreno, S., Ertesvåg, H., Valla, S., Núñez, C., Espin, G., & Cocotl-Yañez, M. (2018). RpoS controls the expression and the transport of the AlgE1-7 epimerases in *Azotobacter vinelandii* . *FEMS microbiology letters* , 365(19), fny210. https://doi.org/10.1093/femsle/fny210

Moreno, S., Nájera, R., Guzmán, J., Soberón-Chávez, G., and Espín, G. (1998). Role of Alternative σ Factor AlgU in Encystment of *Azotobacter vinelandii* . *J. Bacteriol* . 180, 2766–2769.

Núñez, C., León, R., Guzmán, J., Espín, G., and Soberón-Chávez, G. (2000a). Role of *Azotobacter vinelandii* mucA and *mucC* Gene Products in Alginate Production. *J. Bacteriol*. 182, 6550–6556. doi:10.1128/JB.182.23.6550-6556.2000.

Quiroz-Rocha, E., Bonilla-Badía, F., García-Aguilar, V., López-Pliego, L., Serrano-Román, J., Cocotl-Yañez, M., et al. (2017). Two-component system CbrA/CbrB controls alginate production in *Azotobacter vinelandii. Microbiology* , 163, 1105–1115. doi:10.1099/mic.0.000457.

Segura, D., Guzmán, J., & Espín, G. (2003). *Azotobacter vinelandii* mutants that overproduce poly-beta-hydroxybutyrate or alginate. *Applied microbiology and biotechnology* , 63(2), 159–163. https://doi.org/10.1007/s00253-003-1397-1

Steigedal, M., Sletta, H., Moreno, S., Maerk, M., Christensen, B. E., Bjerkan, T., Ellingsen, T. E., Espìn, G., Ertesvåg, H., & Valla, S. (2008). The *Azotobacter vinelandii* AlgE mannuronan C-5-epimerase family is essential for the in vivo control of alginate monomer composition and for functional cyst formation. *Environmental microbiology* , 10(7), 1760–1770. https://doi.org/10.1111/j.1462-2920.2008.01597.x

Trujillo-Roldán, M., Moreno, S., Segura, D., Galindo, E., and Espín, G. (2003). Alginate production by an *Azotobacter vinelandii* mutant unable to produce alginate lyase. *Appl. Microbiol. Biotechnol* . 60, 733–737. doi:10.1007/s00253-002-1173-7.

Vazquez, A., Moreno, S., Guzmán, J., Alvarado, A., & Espín, G. (1999). Transcriptional organization of the *Azotobacter vinelandii algGXLVIFA* genes: characterization of *algF* mutants. Gene, 232(2), 217–222. https://doi.org/10.1016/s0378-1119(99)00119-5
