# Supplementary figures and images for: Genetic Regulation of Alginate Production in Azotobacter vinelandii a Bacterium of Biotechnological Interest: A Mini-Review
Source: Front Microbiol. 2022 Mar 23;13:845473. doi: 10.3389/fmicb.2022.845473 (PMC8988225; doi:10.3389/fmicb.2022.845473)

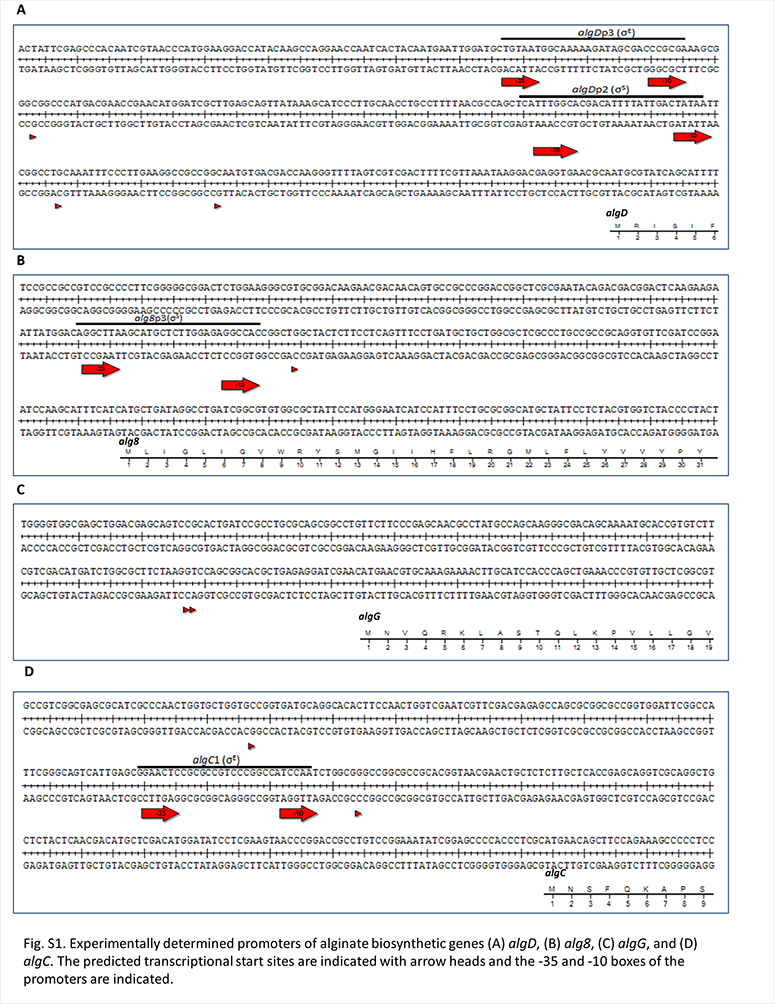

Supplement: Supplementary file 2 [file Image_1.TIF]
